# Response to Letter to the Editor: “Hypertension and Type 2 Diabetes Are Associated With Decreased Inhibition of Dipeptidyl Peptidase-4 by Sitagliptin”

**DOI:** 10.1210/jendso/bvaa006

**Published:** 2020-02-29

**Authors:** Jessica R Wilson, Megan M Shuey, Nancy J Brown, Jessica K Devin

**Affiliations:** 1 Division of Clinical Pharmacology, Vanderbilt Department of Medicine, Nashville, Tennessee; 2 Division of Endocrinology, Diabetes, and Metabolism, Vanderbilt Department of Medicine, Nashville, Tennessee; 3 Division of Endocrinology, Diabetes and Metabolism, University of Pennsylvania Department of Medicine, Philadelphia, Pennsylvania; 4 UCHealth Endocrinology, Yampa Valley Medical Center, Steamboat Springs, Colorado

We appreciate Dr. Taylor’s thorough review of the history of dipeptidyl peptidase-4 (DPP4) activity measurements. Although dilution of sitagliptin could account for the slightly diminished inhibition of DPP4 activity in controls compared with that previously reported, it could not account for the difference between controls and obese hypertensives reported in the paper, as DPP4 activity was measured using the same methodology in all groups. In addition, we reported an inverse correlation between body mass index and DPP4 activity, suggesting the effect may have been related to the volume of distribution of sitagliptin.

## Abbreviation

DPP4: dipeptidyl peptidase-4

## **Additional Information**:

***Disclosure Summary:*** The authors have nothing to disclose.

